# An NIH intramural percubator as a model of academic-industry partnerships: from the beginning of life through the valley of death

**DOI:** 10.1186/1479-5876-9-54

**Published:** 2011-05-08

**Authors:** Michael R Emmert-Buck

**Affiliations:** 1 Maryland, USA

## Abstract

In 2009 the NIH publicly announced five strategic goals for the institutes that included the critical need to translate research discoveries into public benefit at an accelerated pace, with a commitment to find novel ways to engage academic investigators in the process. The emphasis on moving scientific advancements from the laboratory to the clinic is an opportune time to discuss how the NIH intramural program in Bethesda, the largest biomedical research center in the world, can participate in this endeavor. Proposed here for consideration is a percolator-incubator program, a 'percubator' designed to enable NIH intramural investigators to develop new medical interventions as quickly and efficiently as possible.

## Introduction

*I have sometimes thought that, in order to be a good minister, it was necessary to leave the ministry. The profession is antiquated. In an altered age, we worship in the dead forms of our forefathers*.

- Ralph Waldo Emerson

The present proposal is in response to the recent call by the NIH to develop new methods that facilitate the translational research process [1-3]. At the outset, it is imperative to crystallize what is being articulated - not so much a business model or administrative structure but an *environment *where academic percolation and business incubation occur together, back and forth, short-term and long-term, without artificial walls of separation, and where investigators are enabled to use their knowledge to rapidly move discoveries to the public. The percubator is a partnership between government and business that incorporates the best of both to responsibly but urgently translate research findings into clinical interventions. Importantly, the program corrects a fundamental engineering problem present in most translational research systems - inefficient bi-directional movement of individual researchers between academic and commercial silos.

## Discussion

**Figure 1:**
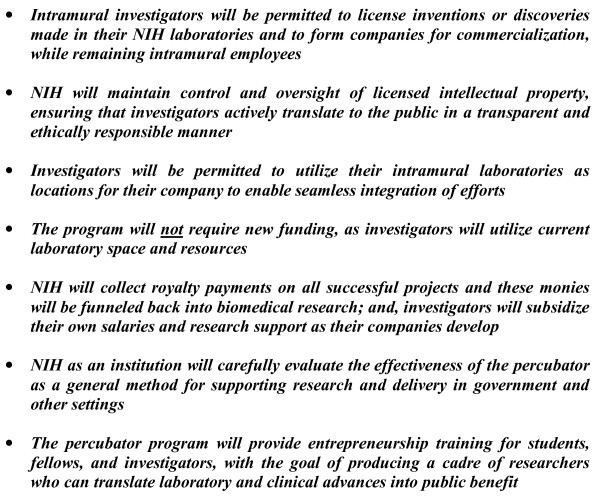
**Elements of an NIH intramural percubator program**.

Conceptually, the program will consist of the seven elements listed in Figure 1. Some might argue a percubator blurs an important distinction between basic science and private sector activities, but this is not accurate. Academia is a scintillating environment where investigators can ponder and question nature irrespective of an immediate utility - it's a percolator of ideas, inventions and biological discoveries, a place where one can 'wonder if and wonder how'. In contrast, companies focus primarily on applied science, taking early concepts from the percolation phase and incubating them through early testing, and then into the competitive world of the marketplace and clinic. The goal of the percubator is *not *to meld these two modes of thinking together; rather, it is to remove the barrier that separates them and allow free and unfettered movement of investigators between the two, anticipating that it will be synergistic to do so and even necessary in some instances, especially considering the arduous path from laboratory to the public. Development of a drug or device is not a linear and unidirectional movement of a single idea, but is an iterative and parallel movement of many related sub-ideas and sub-projects occurring at various points and in different directions along the translational vector. Repetitive cycles must be performed; from percolator to incubator and then back to the drawing board (percolator) again to solve this problem or that. A translational system of 'academia discovers over here and then biotech or pharma commercializes over there' is not necessarily ideal. The percubator will allow these activities to occur in one place, under the long-term and sustained direction of one individual, and will augment the methods that are currently in place.

At present, the barrier between percolation and incubation in the NIH intramural program is often close to impermeable. In contrast, universities and many research institutions contain a semi-permeable barrier that allows academic investigators to work in both arenas, albeit in separate locations, with different people and not always with control over scientific direction once the work 'leaves the lab'. The proposed NIH percubator program pushes this concept one step further by eliminating the barrier altogether, but at the same time respecting and building upon the distinction and uniqueness of academia and the private sector. A likely outcome is that investigators in the program will continue to focus on basic research as a primary function, but when the time is right they will quickly move a discovery to their innovative small company and into early incubation, facilitating testing of more ideas for translational potential than is done now, and then further developing drugs or devices that show promise, all the while overseeing the process with the depth of field that arises naturally from a binocular science-business view. Moreover, participating first-hand will improve investigators' savvy with respect to the needs of the market, which in turn will aid in assessing the translational potential of ideas that are still in the percolation phase.

The NIH intramural program is an ideal test site for such new translational research approaches, percubator or other. Novel systems can be evaluated in an open and transparent manner, with successful methods expanded upon and unsuccessful ones closed out for good cause based on empirical data. Beyond improving the translational efficiency of the intramural program, these controlled experiments may help inform the process more generally, providing valuable information for other government departments, universities, and non-profit institutions [4].

### Direction and Drive

After a new drug or medical device is percolated to life and progresses through the early incubation phase it must then traverse the so-called valley of death - from promise to real delivery - from drug candidate to effective therapeutic - from prototype to useful product. Most translational efforts die here and there are multiple financial, scientific and sociological reasons for this phenomenon [5-8]. To help overcome these difficulties, the new percubator program offers two important elements to facilitate this journey in the intramural program; direction and drive.

#### Direction

Creating effective new therapies, diagnostics or medical devices is a challenging and even audacious objective. With the remarkable complexity of human physiology and related pathogenesis, and the difficulty in rational intervention even when disease etiology is known, it is perhaps not surprising the number of FDA approved agents is dwindling. At the current pace of therapeutic development the on-going transition from 'screening for drugs' to 'science-based design of drugs' may be a century-long endeavor not a decades-long one. Mindful of the enormity of the undertaking and the urgent needs of our patients, the requirement for a highly efficient translational research system becomes apparent.

Enabled by the percubator program, intramural investigators could provide novel and distinctive translational roadmaps related to their diseases-of-interest, a critical contribution given the value of academic discovery in the drug development process [9-11]. As company decision-makers they would be positioned to set direction for moving interventions from laboratory to patient, including original paths-less-traveled based on their expertise and knowledge base. The safe harbor of the percubator will provide both the short-term and long-term resource support to go from start to finish, alone or in a later-stage, de-risked partnership with other companies - down into the valley of death, across the basin, and up the steep grade of the opposite hill. For drug development, the percubator could enhance important activities that are currently endangered by market forces. As one example, integrated academic-commercial scrutiny of biological mechanisms of lead compounds could be an important niche for companies in the percubator to fill when the target protein or pathway is the subject of an investigator's lifelong investigation. As a second example, new medical interventions for rare or neglected diseases would be an excellent outcome of the percubator program. Since the aim is to improve human health and not necessarily to develop a 'blockbuster drug', companies in this uniquely blended academic-private sector environment may feel freer to pursue projects with a less attractive market, especially when they dovetail with investigators' basic science interests.

This latter scenario may be an important feature of the percubator, particularly in light of today's usual translational approach: a) academia hands off an application to the private sector only if and when it meets a threshold of projected revenues; b) new research findings are published in an academic journal with the hope that the information is eventually of use to the private sector and hence the public. Both of these methods are productive and appropriate of course; however, they neglect certain kinds of activity that the percubator could support - for example, a 'high benefit-low profit' translational route - development of tangible products that will advance human health but will generate only modest or minimal financial returns. To become a reality these therapies, devices, or diagnostics will require a novel kind of company to produce them, within a funding environment that supports and values such activities. The recent development and increasing popularity of low-profit limited liability companies (L3Cs), business entities with both a social and an economic agenda, represents new thinking along these lines and could serve as a template for commercialization efforts in the percubator [12,13]. And as a practical matter, designating percubator investigators and their companies as 'contractors and contract companies' would be a simple route to initiating a pilot program in Bethesda as these mechanisms are routinely employed by NIH in support of many academic and commercial activities.

The above being said, a percubator may in fact produce consequences besides the hoped-for improvements in human health - large monetary returns and significant economic development [14,15]. A central premise is that an integrated science-business environment will be uniquely conducive to innovation, thus there is a real possibility that percubator companies will become highly successful businesses, facilitating growth of the biotech sector and returning significant funds to the public to support further biomedical research.

Irrespective of the support mechanism or economic impact, the primary goal of the percubator will be to fully engage investigators in long-term and creative translation of their work to benefit the public: "I think this application has great market potential and is ready for corporate development - that one is best developed through an L3C - those two are going to be important five years from now and need to percolate more - and this one is risky and has a limited market, but I am going to commercialize it anyway, the science is interesting and it could help my patients."

#### Drive

Once direction is established the success of a translational endeavor then shifts to the motivation of the participants involved. Pride of inventorship, a desire to make scientific progress and to be recognized for doing so, and a longing to help patients are strong motivations in themselves, but are not always sufficient for this arduous undertaking. The percubator also harnesses the clarion call to the entrepreneur, one of the most potent forces of the human psyche. Of course, this necessarily brings up thorny issues around financial remuneration and company entanglements that at first glance would seem problematic for such a program in Bethesda, but closer inspection reveals this is not so. The intent of the percubator is for intramural investigators to commercialize their *own work *under the auspices and watchful eye of the NIH as licensor; the proposal does not argue for the types of independent consulting arrangements or other deals with industry that were problematic for the intramural program in the past. The goal is quite simple - integrate wise government investment and scientific expertise with the strong incentives of the free market to urgently deliver the benefits of research to the public. To ignore the value of any motivational element is a disservice to our future patients and it's their health and wellbeing that hangs in the balance. Would we design a translational system *de novo *that prohibits investigators from publishing and receiving academic credit for their work? Of course we would not. Similarly, financial rewards for those who deliver to the public should be in place.

However, money is not the sole driving force of the entrepreneur, in fact it's often of secondary importance and simply a means to an end. To many the 'fire in the belly' here is the creation of new commercial life; seeing, believing in, and growing an entity that meets a need, with the freedom to pursue unique directions and the operational control to turn-on-a-dime as conditions warrant in order to make progress. The process is thrilling, motivating and difficult all in one, much like parenting a young child and nurturing them along. To disregard or diminish this particularly important element within a translational system is imprudent - we do so at the peril of the public we seek to help.

Undoubtedly, many investigators at NIH will have little interest in a percubator. They will continue on making important advances in the laboratory and clinic, wedded to and motivated by their unbridled love for science and desire to make a positive difference, and content to use the conventional translational system. But to other segments of the community the program likely will unleash a torrent of new activities - innovative small corporations; L3Cs; and non-profits - all serving as unique pathways from discovery to patient.

## Conclusion

*To know even one life has breathed easier because you have lived. This is to have succeeded*.

- Ralph Waldo Emerson

In closing, a strategy to enhance translational activities at the NIH is proposed, fully integrating the free thinking mentality of academia with the productive drive of the private sector to ensure that no stone is left unturned in efforts to help the public. A new percubator program, along with the rich intellectual environment and long-term support of science could make Bethesda 'the place to be' for the next generation of translation researchers, helping to ensure that a distinguished past is an equally distinguished future.

## Competing interests

MRE-B is a translational researcher who has participated in many laboratory and clinical research studies, as well as several successful biotechnology company start-ups, both privately and in his current role as a principal investigator in the NIH intramural program. The article was written in his personal capacity and the views do not represent those of the Department of Health and Human Services, National Institutes of Health, or U.S. Public Health Service.

The author declares that he has no competing interests.
